# Density Modulations in Active Colloidal Systems through Orthogonal Propulsion Control and Sensory Delays

**DOI:** 10.1021/acsnano.5c12596

**Published:** 2025-10-27

**Authors:** Ueli Töpfer, Maximilian R. Bailey, Sanjay Schreiber, Federico Paratore, Lucio Isa

**Affiliations:** Department of Materials, 27219 ETH Zurich, 8093 Zurich, Switzerland

**Keywords:** active matter, density modulations, sensory delay, orthogonal control, induced-charge electrophoresis, photoconductivity

## Abstract

Recent advancements in active colloidal systems aim to mimic key characteristics of biological microswimmers, particularly their adaptive motility in response to environmental changes. While many approaches rely on externally imposing a variable propulsive force, achieving true autonomous and self-regulating adaptation to the environment remains limited. In this study, we take a step in this direction and develop Janus microswimmers driven by electrohydrodynamic flows that autonomously adjust their propulsion dynamics in response to varying illumination and exposure to chemical agents. Our Janus particles are silica colloids partially coated with titania, which self-propel via induced-charge electrophoresis under uniform AC electric fields. Since titania is photoconductive, it increases its conductivity under UV illumination, which thereby regulates the propulsion velocity independent of and orthogonally to the applied electric field. Crucially, the velocity adaptation happens spontaneously but requires a finite amount of time. This sensory delay leads to enhanced microswimmer localization in response to spatiotemporal light modulations compared with the typical case of instantaneous response considered for synthetic microswimmers. Additionally, the particles spontaneously adapt their response time in the presence of chemicals, here methanol, which affect the lifetime of charge carriers and lead to a concentration-dependent response. By harnessing these dynamics, akin to those of biological microswimmers, we control both local and global particle behavior, presenting exciting opportunities for adaptive active matter systems.

## Graphical abstract

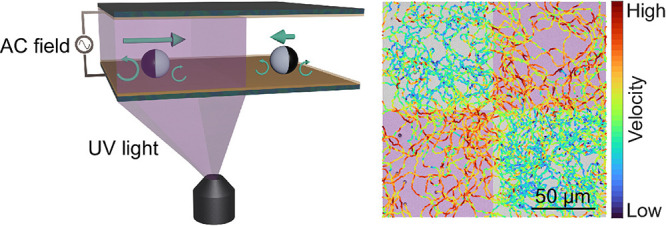

## Introduction

Spontaneous spatial and temporal variations of population density in response to environmental inputs are central to life at all length scales, from survival strategies in populations of complex organisms to embryonic development and biofilm formation in cellular colonies. The ability to sense, elaborate, and actively respond to stimuli and signals, from visual cues to light or chemical fields, is an essential feature of adaptive behavior where individual units regulate their functions and thus contribute to coordinated collective responses. At the microscale, some motile organisms are capable of adapting their motility in response to chemical fields^1^ in order to accumulate in regions of high nutrient concentration,^2^ to proliferate in areas with defined oxygen levels^3,4^ or to actively seek natural light.^5,6^

In synthetic microscale active systems, such as ensembles of self-propelling Janus particles, local density modulations can be used to template materials,^7^ assemble small-scale devices,^8,9^ regulate flows,^10^ and carry out complex manipulation tasks.^11,12^ While physical interactions can lead to structure formation^13^ and density variations, i.e., including motility-induced phase separation,^14,15^ density variations can also spontaneously emerge as a consequence of spatial motility modulations even in the absence of specific interactions among the active units.

A general theoretical result in fact determines that $\rho \left(r\right)\propto \frac{1}{v\left(r\right)}$ , where ρ­(*r*) is the steady-state mean number density of microswimmers at position *r* and *v*(*r*) is the corresponding local propulsion speed.^16–18^ The result is exact for noninteracting objects with a position-dependent swimming speed that is independent of local density, with corrections to account for those effects. Nonetheless, this description makes the crucial assumption that the velocity changes instantaneously as a function of the position. While this condition may be implemented in numerical simulations,^17^ any real system will have a finite response time.

This velocity adaptation can then be described by introducing a response time, τ, defined as the characteristic time to update propulsion speed in response to sensing an input. In most experimental realizations of artificial microswimmers with propulsion control, τ is assumed to be negligible. This assumption holds if two conditions are verified: (i) τ ≪ *D*_R_^–1^, where *D*_R_^–1^ represents the characteristic Brownian reorientation time, implying that the direction of motion does not change before the velocity *v* is updated, and (ii) τ *v* ≪ *R* implying that the swimmer travels a small distance compared to its radius *R* during the time τ, which is the typical case.^19–21^ However, there are few exceptions in which delayed responses are deliberately engineered to alter steady-state swimmer density distributions.^22^

In contrast, biological microswimmers often exhibit significantly longer response times due to the involvement of complex biochemical signaling pathways that lead to velocity adaptations. As a result, the distance traveled before the velocity changes, τ*v*, frequently exceeds the swimmer size (τ*v* > *R*), and the response time may be either shorter or longer than the reorientation timescale.^23,24^ An intermediate and highly tunable case is that of macroscopic robots, where response parameters can be fully programmed. In such systems, the distance traveled during velocity adaptation typically exceeds the size of the robot (τ*v* > *R*), while the relation to the rotational timescale can be freely adjusted to either suppress or enhance density modulations, depending on the desired behavior.^25,26^

In principle, density variations can be derived from any mechanism providing local propulsion control. Nonetheless, in practice, light-controlled microswimmers have emerged as an optimal solution due to the ease of spatial and temporal modulation of light intensity patterns via digital projection devices and spatial light modulators. Light typically acts as the stimulus responsible for the propulsion, e.g., by driving ionic fluxes across the cell wall of motile bacteria^27^ or due to photothermal effects, either directly via temperature gradients^28–30^ or by coupling those to chemical^31^ or surface tension gradients.^32^ Therefore, in these cases, light is the sole control parameter in the system. Having a single control parameter limits both the versatility and the robustness of applicable strategies to regulate motility and, correspondingly, density modulations within independently available environmental energy sources. An exception may be the case of photocatalytic microswimmers,^33–35^ where the particles actively decompose environmentally available chemical fuel at an illumination-dependent rate. However, independently and dynamically controlling fuel concentration levels is challenging.

A preferred alternative would instead require the realization of systems with propulsion control mechanisms orthogonal to the mechanism generating it, very much like a sailboat that can regulate speed independently of the wind by changing the surface of its sails. Whereas examples exist in biological swimmers, for instance, positive chemotactic response toward nonmetabolizable attractants,^36,37^ or gravitaxis observed under constant nutrient conditions,^38^ strategies for orthogonal propulsion control in synthetic systems are far less frequent. In particular, shape reconfiguration^39–41^ and external regulation of material properties,^20^ or both,^42^ have been proposed in the literature as single-particle-level solutions, opening up exciting opportunities to implement new strategies for global control of particle density.

In this work, we report a strategy to regulate the spatial distribution of photoresponsive silica–titania Janus microparticles powered by uniform AC electric fields via the orthogonal control of the conductivity of the titania caps through UV illumination. We discuss the resolution of particle localization or patterning as a function of the sensory delay imposed by the properties of the titania cap. We furthermore show that this sensory delay depends on the concentration of methanol present in the sample, demonstrating that our particles spontaneously adapt their dynamics in response to local chemical cues. From these results, we draw guidelines to dynamically engineer the localization of synthetic microswimmers by the selection of materials with tailored responses.

## Results and Discussion

### Propulsion Adaptation

Our particles are fabricated from silica colloids of 2.06 μm diameter that are half-coated with a 50 nm-thick layer of titania (Figure 1a). These Janus particles exhibit self-propulsion through induced-charge electrophoresis (ICEP),^43,44^ which is based on the contrast in conductivity between the coated and bare hemisphere.^45^ The titania, in its anatase phase, exhibits photoconductivity, implying that its electrical conductivity increases upon illumination with photons of wavelengths corresponding to the energy of the bandgap or higher (λ ≤ 387 nm),^46,47^ thus enabling control over the conductivity contrast of the Janus particles via UV illumination.^20^ As shown in Figure 1a, the particles are confined in a liquid layer formed between two parallel and transparent electrodes and, due to gravity, settle onto the bottom one. Upon applying an alternating (AC) electric field in the kHz frequency range, ICEP induces an asymmetric flow field around each particle that propels them in a direction parallel to the electrode surface. The particles move at a constant height above the substrate so that any wall-related effects on ICEP propulsion are expected to remain constant during motion.^43,48^ As expected for ICEP, we measure swimming velocities proportional to the square of the applied electric field, given as $v\propto {\left(\frac{V_{\mathrm{pp}}}{h}\right)}^{2}$ ,^49^ as shown in Figure S1. Here, *V*_pp_ represents the amplitude of the applied voltage (varied experimentally between 1 to 10 V) and *h* is the electrode spacing (fixed to 120 μm in our setup).

**Figure 1:**
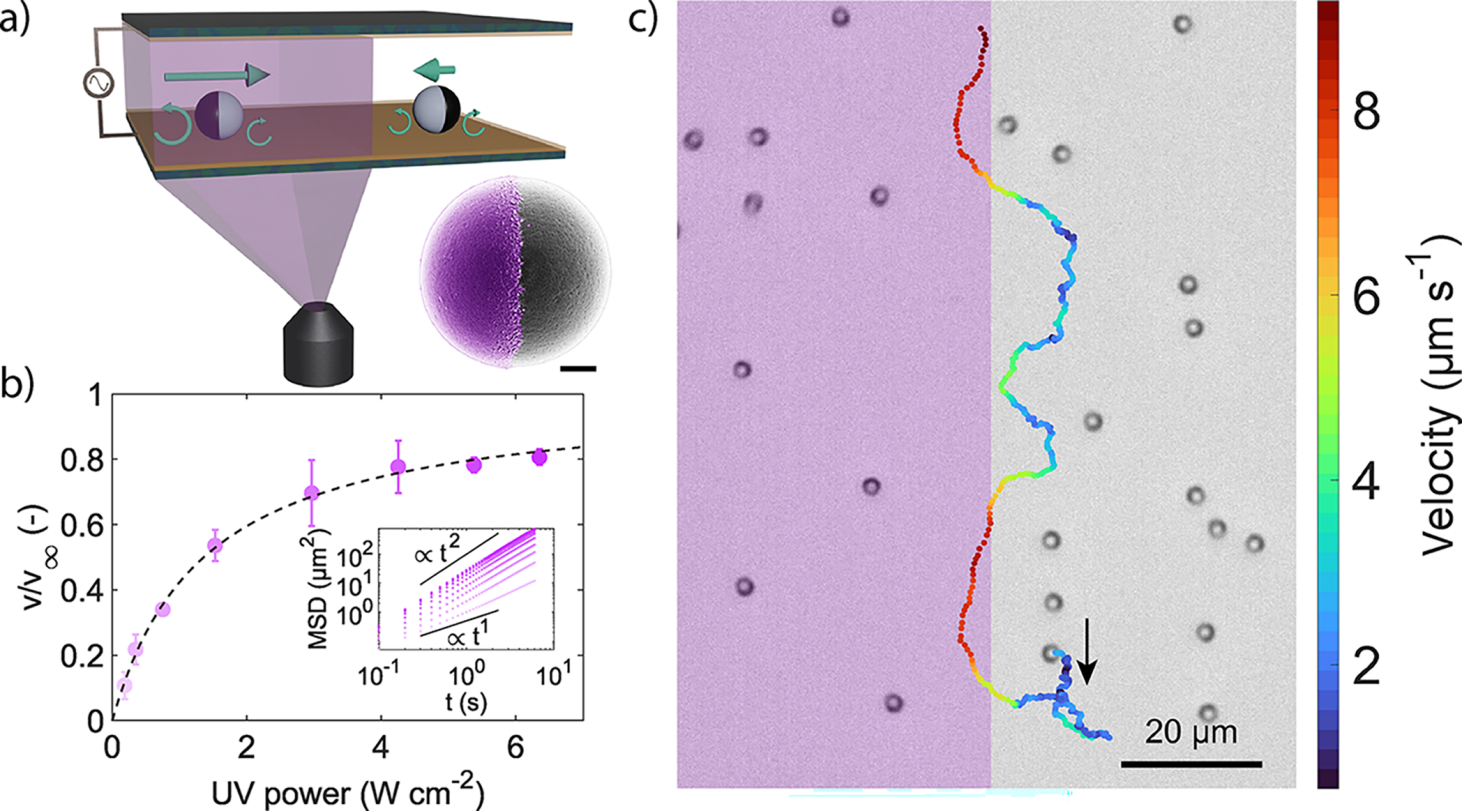
Adapting active particle velocity via light modulation. (a) Schematic of an experimental cell illustrating the setup for our experiments. The curled green arrows represent the electrohydrodynamic flows around the Janus particles (with the titania-coated hemisphere shown in black), which are modulated by the UV light pattern projected through the microscope objective via a digital micro-mirror device (DMD). Inset: scanning electron micrograph of an individual Janus particle with the titania cap false-colored in violet. The scale bar corresponds to 500 nm. (b) Particle velocity, *v,* (5 V and 4 kHz) normalized by its saturation value, *v*_*∞,*_ as a function of UV light intensity. Saturation velocities were obtained from three independent particle batches and experimental cells, yielding values of *v*_*∞*_ = 3.4, 5.5, and 5.7 μm s^–1^. Inset: ensemble-averaged MSD curves for light intensity levels corresponding to the colors of the data points in the main graph. (c) Optical micrograph and particle trajectory (10 V and 10 kHz) in a binary on–off stripe illumination pattern (illuminated area colored in violet; 6.3 W cm^–2^). The color bar represents the particle velocity, calculated from the displacements over 300 ms.

We control particle velocity *v* by modulating the illumination intensity within the cell using light with a wavelength of 365 nm and a power density ranging from 0 to 6.3 W cm^–2^. As displayed in Figure 1b, for fixed electric fields (5 V at 4 kHz), the normalized velocity first increases linearly with increasing intensity before asymptotically approaching a plateau value (*v*_*∞*_) at higher intensities. This follows a Michaelis–Menten-like kinetic, as previously reported for the propulsion velocity of photocatalytic microswimmers.^7,34,50^ We extract these active velocities from ensemble-averaged mean-squared displacements (MSD) of Janus particles subjected to globally uniform illumination of varying intensities (Figure 1b inset), following the approach outlined in the Methods section.

Opposed to systems where titania photocatalytically decomposes chemical fuel to generate propulsion,^33–35^ we assume such reactions to be negligible in our system. Any significant asymmetric chemical reaction^51^ or electrochemical process^52^ would otherwise result in self-diffusiophoretic or self-electrophoretic motion in the absence an of applied electric field under UV illumination. However, as shown in Figure S1, the particle velocity decays to zero under full UV illumination when the applied electric field is reduced, confirming the absence of such effects in our system.

Using a digital micromirror device (DMD) placed within the optical path of the microscope, we then project light patterns across the field of view, thus creating tailored spatiotemporal modulations of the light intensity in which the particles move. Figure 1c presents a typical trajectory of a particle navigating a binary stripe illumination pattern, alternating between the 0 (gray) and 6.3 W cm^–2^ (violet) regions. The particle’s velocity varies significantly, from less than 1 μm s^–1^ in dark areas to greater than 8 μm s^–1^ in illuminated regions. Correspondingly, its persistence length, *L*_p_ = *v*/*D*_R_, which is the average distance a particle moves before decorrelating its orientation due to its rotational diffusivity *D*_R_, is substantially higher upon UV illumination. However, from the qualitative color change in the trajectory, one observes that the velocity adaptation is not instantaneous upon crossing the illumination boundary.

### Sensory Delay

To investigate the time dependence of the velocity adaptation to changes in illumination, we therefore subjected the particles to cycles of ON and OFF illumination, i.e., switching the illumination intensity between 6.3 and 0 W cm^–2^ (Video S1). Figure 2a shows a typical particle trajectory during such a cycle, where we observe the persistence of a higher propulsion speed after the UV light is turned off and a rapid acceleration upon turning it on again. We then quantified this response by measuring the ensemble-averaged velocity for all particles in the sample. Figure 2b reveals a strong asymmetry in the response times: the particles exhibit a finite delay when transitioning from high to low activity, while they immediately adapt from low to high activity.

**Figure 2:**
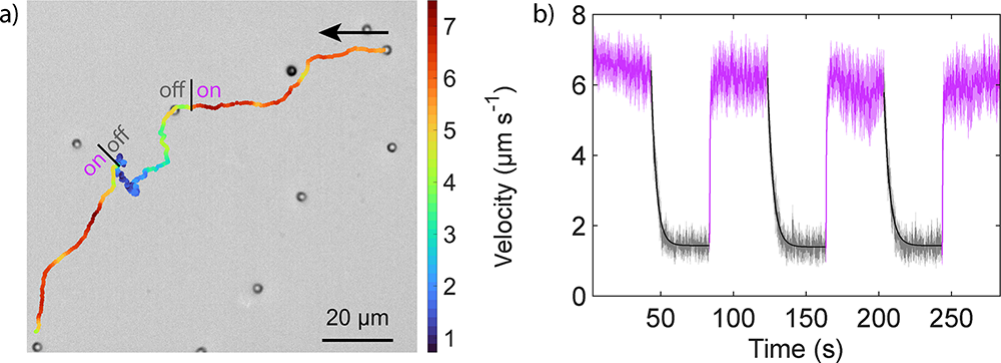
Finite sensory delay. (a) Optical micrograph showing the trajectory of a single particle undergoing a cyclic switch of the global illumination from 6.3 to 0 W cm^–2^ and back to 6.3 W cm^–2^ (with *V*_pp_ = 5 V at 4 kHz). The color bar represents the particle velocity, calculated from the displacement over 300 ms. (b) Ensemble-averaged swimming velocity of 144 particles subjected to multiple cyclic changes of the global illumination (with *V*_pp_ = 5 V at 4 kHz). The black lines represent exponential fits to the velocity transitions between the ON and OFF illumination states (eq 1). The velocities were calculated from the displacement over 300 ms and averaged across the particle ensemble, with the shaded area representing the standard deviation.

We quantified these adaptation timescales by fitting the change in velocity with an exponential decay function:

$$v\left(t\right)=v_{i}+\left(v_{i-1}-v_{i}\right)e^{-t/\tau }$$ 1

In this equation, *v*(*t*) is the velocity at a given time *t*, and *v*_*i*–1_ and *v*_*i*_ are the velocities at the end of the previous illuminated and current dark phases, calculated as the average velocity over 20 s, respectively. τ is the characteristic response time, which is the sole fitting parameter. This quantification reveals that the response time to slow down is on the order of 2 s. The response to increasing illumination appears effectively instantaneous, as any delay shorter than a few hundred milliseconds falls below the temporal resolution of the velocity measurement (300 ms displacement window). Delays on the order of seconds are usually not explicitly considered in photoactive colloidal systems and are often treated as instantaneous,^53^ even if, as we will see later, have deep implications in defining the steady-state distribution of particles under patterned illumination.

We attribute the slow decay of the velocity when the UV light is turned off to the persistence of charge carriers in the titania cap over some finite time. While charge carrier lifetimes on the order of seconds are unusually long for semiconducting materials, studies have reported photogenerated electron lifetimes ranging from seconds to minutes. These extended lifetimes are attributed to the stabilization of surface-trapped holes through hydrogen-bonding interactions between adsorbed water and TiO_2_.^54,55^ This response is very different from the reaction of the particles to switching on and off the AC electric field while the illumination intensity is kept constant, where the adaptation is symmetric and instantaneous (Figure S2).

Consequently, we hypothesize that adjusting the surface redox kinetics offers a direct means to modulate the adaptation timescale upon illumination removal. One effective strategy is the introduction of sacrificial hole scavengers such as alcohols. Methanol, in particular, reacts readily with photogenerated holes, suppressing electron–hole recombination and thereby extending the effective lifetime of photoexcited electrons on the particle surface.^56,57^ To investigate this effect, we exposed the particles to varying concentrations of methanol and monitored their velocity response during repeated ON/OFF cycles (Figure 3). Remarkably, by adding 0.5 M of methanol to the system, the characteristic lifetime can be increased by a factor of almost four. On the other hand, the timescale over which particles reaccelerate upon turning the illumination on remains unaffected (see Figure S3). Importantly, the asymmetric decomposition of methanol does not lead to any significant self-phoretic motion under UV illumination and without the AC field, as shown in Figure S4. The ensemble-averaged MSD under full illumination follows a linear relation with a diffusion coefficient of *D*_T_ = 0.11 μm^2^ s^–1^, consistent with experiments performed without methanol. The data show that our microswimmers autonomously regulate their dynamics in the presence of chemical cues and that environmental factors affecting their electrical properties couple into their self-propulsion.

**Figure 3:**
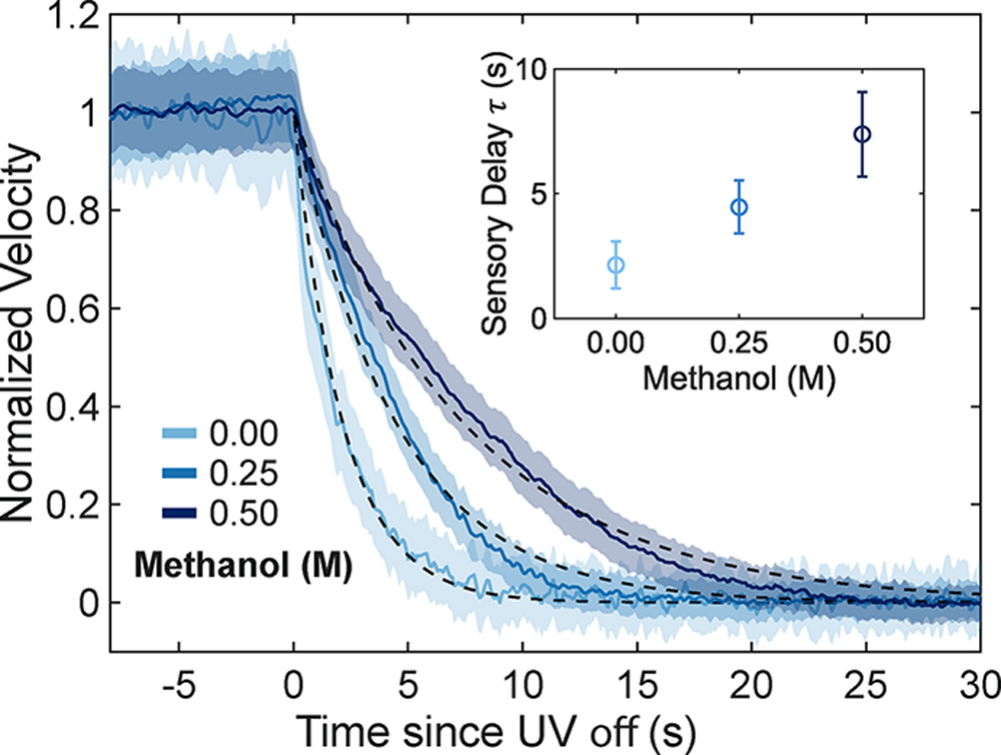
Influence of methanol on sensory delay. Mean swimming velocity of particles in pure water (no methanol) and in solutions containing 0.25 and 0.5 M methanol, showing the delayed decrease in velocity following the switch from ON to OFF illumination. For each condition, velocities were first ensemble-averaged for each measurement and then averaged across three measurements per cell from at least three separate cells and particle batches. All experiments were performed at a frequency of 4 kHz, with the voltage amplitude *V*_pp_ adjusted to ensure a pronounced velocity difference between the illuminated (ON) and nonilluminated (OFF) states. Velocities were calculated from particle displacements over 300 ms. The shaded area represents the standard deviation obtained via error propagation across the individual measurements. Black lines correspond to exponential fits of these transitions, obtained using eq 1. The inset shows the mean and standard deviation of the fitted decay times for each condition.

### Localization

We investigate the combined effect of spatial and temporal modulation of particle motility via light signals as a mechanism to control the global density distribution of particles.^58,59^ Using a DMD, we project checkerboard patterns of ON (6.3 W cm^–2^ – violet) and OFF (gray) regions of characteristic size *L* corresponding to regions of high and low particles’ activity, respectively (Video S2). The top row of Figure 4 shows experimental trajectories, color-coded by velocity, for particles moving through patterns with increasing *L* from 20 to 80 μm.

**Figure 4:**
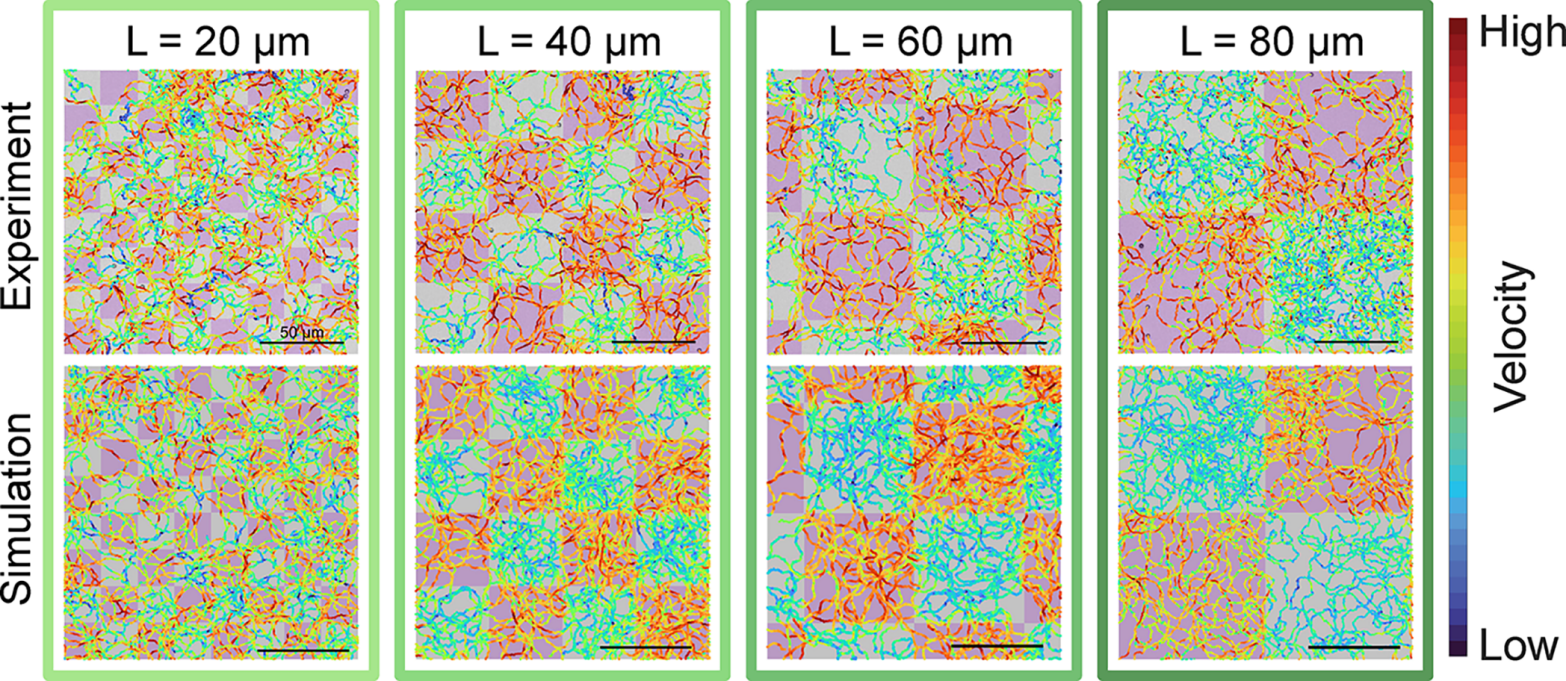
Particle motion on checkerboard motility patterns. Top row: experimental particle trajectories within illumination checkerboard patterns (0 W cm^–2^–gray and 6.3 W cm^–2^–violet) with varying size *L* at 4 V and 4 kHz in media without any added methanol. Bottom row: simulated particle trajectories with input parameters were extracted from the corresponding experiments. Instantaneous velocities are color-coded between *v*_L_ and *v*_H_.

In order to ensure that we have a conceptual understanding of the crucial factors at play, we replicate the experimental conditions using a numerical simulation framework previously developed for spatial modulations of rotational dynamics^22^ and active velocity.^60^ In brief, the simulation solves the Langevin equations of motion for noninteracting particles with position-dependent velocity. Specifically, each particle has a defined minimum (low) and maximum velocity (high), *v*_L_ and *v*_H_, respectively, and switches from high to low at a rate proportional to $\frac{1}{\tau }$ , where τ is the characteristic response time (more details in the Methods section). The bottom row of Figure 4 shows simulated trajectories obtained using the experimentally extracted values of *v*_L_, *v*_H_, and τ, with color coding following the experimental procedure (see also Video S3).

We quantify the particle number density in nonilluminated areas ρ_L_ and illuminated ones ρ_H_ by counting the particles in the respective regions in every frame. As initially described, at steady state, we expect to see a localization of particles in lower-velocity regions according to the theoretical predictions.^14,16,17^ We quantify the degree of localization as the ratio between ρ_L_ and ρ_H_, with ρ_L_/ρ_H_ = 1 corresponding to no localization. For systems with velocity adaptation, we thus expect localization to follow the simple relation ρ_L_v_L_ = ρ_H_v_H_, where *v*_L_ and *v*_H_ are the two corresponding swimming speeds. In our experiments and simulations, we indeed recover this proportionality for small pattern sizes (Figure 5). However, upon increasing the velocity ratio *v*_H_/*v*_L_ and the pattern size *L*, we achieve an “over-localization” of particles in the low-motility regions, effectively allowing for a more efficient accumulation of the particles within the slow areas. In order to further elucidate how this behavior depends on the parameters of the systems and, in particular, on the response time τ, we then carried out a set of systematic numerical simulations.

**Figure 5:**
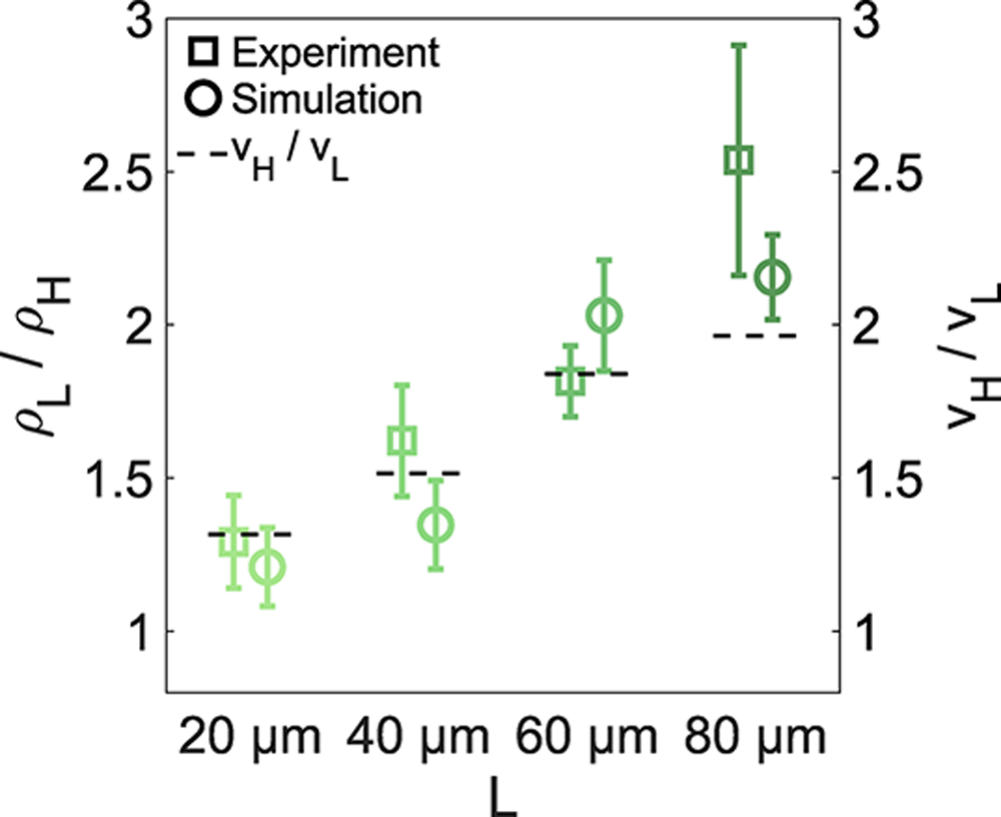
Quantification of particle localization. Comparison of the localization, defined as the ratio of particle density in the low- and high-activity areas ρ_L_/ρ_H_, between the experiments (squares) and simulations (circles) for different pattern sizes and velocity ratios. Dashed lines represent the experimentally determined velocity ratios *v*_H_/*v*_L_, which were used as input parameters for numerical simulations.

Figure 6a shows the density ratio as a function of sensory delay for a fixed minimum (*v*_L_) and maximum velocity (*v*_H_) of 1 and 10 μm s^–1^ for different values of *L*. In particular, for *L* = 20 to 40 μm, we start observing a nonmonotonic relation between the response time τ and the localization ratio ρ_L_/ρ_H_. While for fast enough response timescales τ ≲ 0.1 s, the expected localization relation ρ_L_ *v*_L_ = ρ_H_ *v*_H_ is recovered for all values of *L*, localization is significantly enhanced for *L* ≥ 40 μm at an optimal delay time for which ρ_L_/ρ_H_ reaches a maximum. This maximum, as well as the dependence on *L* can be qualitatively understood using simple scaling arguments. Ballistic particles, when transitioning from *v*_H_ to *v*_L_ regions, can penetrate into the low-motility region by a length corresponding to min­(*v*_H_τ, *v*_H_/*D*_R_) that is limited by the smallest of two timescales: the delay time τ and the Brownian rotational time 1/*D*_R_ (for the investigated experimental system τ < 1/*D*_R_). The deeper the particles move ballistically into the low-motility region, the longer it takes them to leave with the low velocity *v*_L_. This sensory delay thus causes the emergence of an optimal delay time, τ*, which increases with increasing pattern size *L*. If the delay is further increased, the localization starts to drop as the particles cannot adapt to velocity changes on a length scale *L* and velocity modulations become decoupled from the underlying motility landscape.^22^

**Figure 6:**
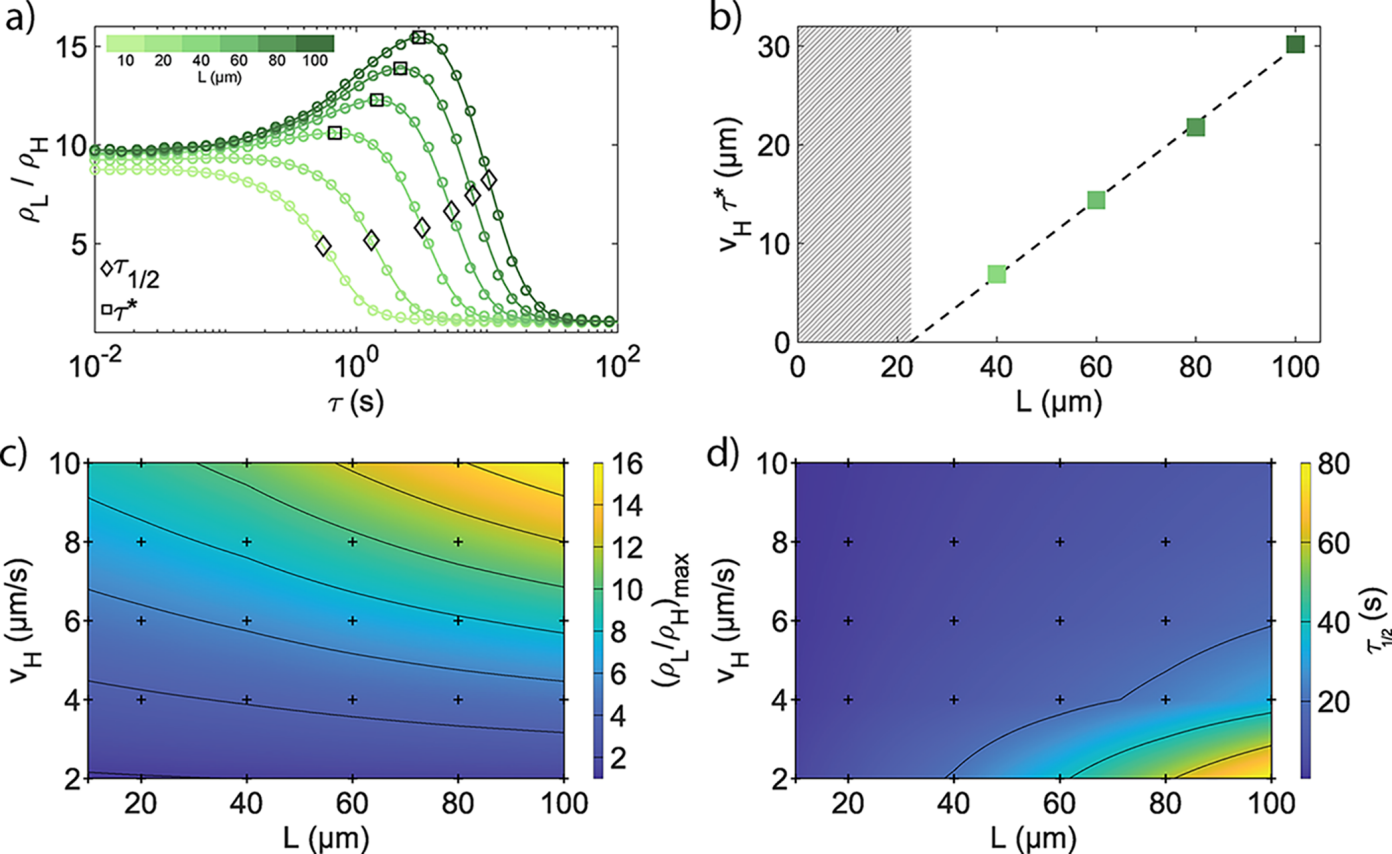
Contrast and resolution in particle patterns from localization. (a) Localization ρ_L_/ρ_H_ as a function of sensory delay τ for checkerboard patterns with sizes ranging from *L* = 10 to 100 μm. *v*_L_ and *v*_H_ are set to 1 and 10 μm s^–1^, respectively. Continuous lines represent smoothing splines fitted to individual simulations (colored circles). We extract two characteristic parameters from each simulation: (ρ_L_/ρ_H_)_max_, the maximum localization obtained at the optimal sensory delay τ* for each *L* (squares), and τ_1/2_, the delay time τ at which localization efficiency drops below 50% of the maximum (diamonds). (b) Optimal adaptation length scale, *v*_H_τ*, as a function of pattern size *L*. The dashed line represents a linear fit to data points for *L* = 40 to 100 μm, and the hatched area shows where the optimal delay drops below 0 (τ* < 0). (c) Map of (ρ_L_/ρ_H_)_max_ for *L* = 10 to 100 μm and *v*_H_ = 2 to 10 μm s^–1^. The continuous map is interpolated between data points obtained from individual simulations (black dots). (d) Map of τ_1/2_ for the same parameters as in c.

We analyze the dependence of the optimal delay time on the pattern size by comparing the traveled distance before adaptation at τ*, *v*_H_τ*, to pattern size *L* (Figure 6b). For large enough patterns *L* ≥ 40 μm, this distance increases linearly with the pattern size, as indicated by the linear regression. This dependence can be quantified by computing the average length *L* ^eff^ of a ballistic trajectory crossing a square domain of size *L*. The calculation requires integrating over all possible trajectories that enter on one side of the square and exit through one of the other three (see section S5). This integration gives *L* ^eff^ ≈ 0.82*L*. Correspondingly, localization is maximized for a penetration inside the low-motility regions of 0.5 *L* ^eff^ ≈ 0.41 *L*, close to the fitted slope of 0.39 in Figure 6b. Thus, this optimal penetration distance follows *v*_*H*_τ* = 0.5­(*L* ^eff^ – *L*_τ*=0_^eff^) ≈ 0.41­(*L* – *L*_τ*=0_), where *L*_τ*=0_ denotes the smallest pattern size for which a finite sensory delay enhances localization (i.e., when τ* > 0). This threshold size corresponds to the persistence length of the particle in the slow region, *v*_L_τ_R_, which represents the ballistic escape distance beyond which delayed adaptation no longer provides a benefit. In our case, *L*_τ*=0_^eff^ = 2 *v*_L_ τ_R_ and thus *L*_τ*=0_ ≈ 14 μm, which is somewhat lower than the fitted value of 23 μm.

Previous work on photokinetic bacteria showed that illumination patterns can be used to control density distributions creating bacterial “images” via motility control.^23,24^ In the spirit of providing quantitative guidelines for pattern optimization for our synthetic microswimmers, we identify two parameters for every given combination of minimum and maximum velocity and length scale, which effectively correspond to a maximum contrast in the pattern and to a delay time above which only 50% of the maximum contrast is achieved for a given pixel size *L*. To this end, we simulated a range of systems with *v*_H_ = 2 to 10 μm s^–1^, *L* = 10 to 100 μm, and fixed values of *v*_L_ = 1 μm s^–1^. The switching timescale τ was varied from 10 to 10^2^ s using logarithmically spaced values. The first parameter is, in fact, the maximum achievable localization (ρ_L_/ρ_H_)_max_. From the data in Figure 6a, we observe that maximum contrast is achieved for instantaneous adaptation at small pixel sizes but takes place at finite values of τ for a larger *L*. Figure 6c thus shows how much the contrast can be enhanced at a given *L* by choosing the optimum sensory delay τ*, demonstrating the existence of an interplay between maximum contrast and pixel size as a function of the particle response time. The second parameter is τ_1/2_, which is the sensory delay above which localization drops below half of its maximum (Figure 6a). As we show in Figure 6d, τ_1/2_ remains below 20 s for a broad range of values and only significantly increases for a combination of low *v*_H_ and large *L* as only a combination of the two increases the residence time of the particles in a given area of the pattern (Figure 6d). These two parameters can thus be applied to govern efficient localization in an active colloidal system, as they define an achievable contrast ((ρ_L_/ρ_H_)_max_) and pixel size (*L*) at which this contrast can be obtained, given a minimum and maximum velocity and as a function of the sensory delay. Their identification thus guides the search for materials with a prescribed value of τ or clarifies which contrast and spatial resolution can be achieved for a given system. The experiments reported in Figure 4 already show that the value of τ can be increased in the presence of methanol, correspondingly increasing the pixel size for optimal localization and the “over-localization.” We can similarly expect that by changing the photosensitive material, the sensory delay can be similarly tuned. We furthermore recall that the presence of an optimal delay time for maximum localization competes with the particle’s Brownian reorientation time, so that the effects of sensory delays can be maximized for larger colloidal particles, given that τ_R_ ∝ *R* ^–3^. We further numerically investigate the evolution of enhanced localization observed at larger delays, i.e., similar to those achieved in the presence of high methanol concentrations, for bigger colloids across different pattern sizes and velocities in the Supporting Information (Figure S12). Even though this regime is beyond the accessible range of parameters, especially in terms of *v*_H_, it nonetheless indicates that a greater localization enhancement can be reached for faster particles.

## Conclusions

Our results demonstrate that adaptive motility in synthetic active colloids can be achieved by incorporating materials that intrinsically modulate their properties in response to environmental stimuli, decoupled from the driving mechanism for propulsion. In our case, while both the AC electric field and the illumination are externally applied, the propulsion adaptation is an intrinsic response of the microswimmers, dictated by the choice of materials and the presence of given chemicals. Synthetic microswimmers incorporating responsive materials thus enable the manifestation of a form of physical intelligence.^61^ This concept implies that functions such as sensing, adaptation, and response, which are digitally programmed in robotic agents or biologically encoded in microorganisms, are transferred to the intrinsic physical properties of the materials that compose the microswimmers.

The use of light to modulate motility, combined with a DMD, for instance, allows independent control of numerous particles at the microscale, enabling mimicking behaviors of biological microswimmers, such as taxis.^62–66^ Moreover, real-time tracking and the use of feedback loops based on particle positions open the door to the development of complex control strategies. On the one hand, those can be used to obtain dynamically self-organized systems to perform coordinated tasks^11,19,67,68^ and to optimize navigation strategies.^69–72^ Conversely, one can implement feedback strategies inspired by natural behavior to introduce effective signaling interactions, where the motility of certain particles in the system is influenced by the behavior of others. Among those cases, visual perception models can be implemented^19^ or, in the future, local particle accumulation or depletion may be triggered upon reaching a given density, similar to the quorum sensing behavior of certain microorganisms.^73^

Looking forward, the integration of dynamic responses in the development of control strategies for microscale active systems will present exciting opportunities. The presence of finite, characteristic adaptation times has already been used to enhance contrast in microswimmer patterns,^24^ to rectify bacterial motion,^74^ to control robotic swarms,^25,26^ and control the polarization of active colloids.^30^ References 25, 26, and 30 also provide a theoretical framework that take into account the effect of delay on the particle density in a one-dimensional system. While the extent of enhanced localization goes up to 20 to 50% in our experiments and simulations, respectively, we remark that these values are set by the physics of active Brownian particles and, in particular, by the competition between the delay time and the intrinsic timescales for reorientation due to Brownian fluctuations. Further enhancement could be realized by considering the case of more persistent swimmers.

The choice of responsive materials may be extended to other cues beyond light, such as temperature, chemical concentration, or pH,^75^ to perform complex tasks in diverse environments^76,77^ in an increasingly more autonomous fashion. Finally, the presence of finite delays for particles to slow down upon removing UV light presents analogies with underactuated control systems, where dynamics may continue after cutting off an energy input. This behavior is suggestive of analogies with what is known as passive dynamics in robotics, which constitutes an important family of control strategies to optimize navigation and energy consumption.^78^

In conclusion, we envisage the rapid evolution of adaptive microscale systems where the convergence of materials’ selection and discovery with control systems will pave the way to the development of colloidal materials with precise microscale control and localization without external computerized intervention.

## Methods

### Particle Fabrication

For the fabrication of the adaptive silica–titania Janus particles, silica colloids (2.06 μm diameter, 5% w/v, microParticles GmbH, Germany) are first diluted to 0.5% w/v in Milli-Q water. Following this, 100 μL of the sonicated and diluted colloidal suspension is spread on a glass slide that was previously cleaned using a 1 min air-plasma treatment. The suspension is then allowed to dry to form a colloidal monolayer. The monolayer is then coated with 50 nm of Ti_3_O_5_ using an e-beam evaporator (Plassys MEB700SL) and subsequently annealed at 500 °C for 120 min under a 1 bar O_2_ atmosphere in a rapid thermal processor (AS-One 150, ANNEALSYS) to ensure a high degree of oxidation of the Ti and to promote its transformation to the TiO_2_ anatase phase. Finally, the particles are retrieved from the slides by sonication in Milli-Q water. The formation of a majority of anatase-phase TiO_2_ was confirmed through X-ray powder diffraction (Figure S6). Prior to an experiment, the particle dispersion is mixed in equal amounts with a 0.2% w/v Pluronic F-127 solution to render a particle dispersion with 0.1% w/v of the surfactant. This surfactant reduces the adhesion of the particles to the substrate of the cell.

### Cell Preparation

The transparent conductive electrodes (16 mm × 16 mm) consist of indium–tin oxide coated glass (<7 Ω sq^–1^, präzisions glas & optik). Two of these electrodes are sandwiched together with a 120 μm thick adhesive spacer with a 9 mm circular opening (Grace Bio-Laboratories SecureSeal, USA). The spacer was halved to provide an in- and outlet through which 9.5 μL of the particle suspension is filled into the cavity before being closed off with grease (Krytox GPL205). The two electrodes are then contacted with adhesive copper tape and connected to a signal generator (33522A Arbitrary Waveform Generator, Agilent) that applies the AC electric field with varying voltages between 1 and 10 V at frequency between 4 and 10 kHz.

### Experiments

All experiments are conducted on an inverted optical microscope (Eclipse Ti2-E, Nikon) with magnifications of 40x (CFI S Plan Fluor ELWD 40XC, Nikon, Japan) and 60x (CFI S Plan Fluor ELWD 60XC, Nikon, Japan). The intensity of the UV light (λ = 365 nm, UHP-F-365, Prizmatix) can be varied from 0 to 6.3 W cm^–2^. The illumination is patterned within the microscope’s field of view using a DMD pattern illuminator (Polygon 1000, Mightex, USA). A DAPI filter cube is used to remove most of the reflected UV light and avoid oversaturation of the camera. Image sequences (2160 × 2560 pixels) of various lengths are obtained with an sCMOS camera (Andor Zyla) at 10 fps. The particle positions in each frame are detected and linked to trajectories using various custom-built Matlab routines.

The translational diffusion coefficient *D*_T_ for each experimental cell was obtained by fitting the ensemble-averaged MSD in the absence of an applied electric field with the theoretical MSD for a particle undergoing Brownian motion:

$$\mathrm{MSD}\left(\Delta t\right)=4D_{T}\Delta t$$ 2

Active particle velocities were obtained by fitting ensemble-averaged MSD curves with the theoretical MSD model for active particles given by^51,79^

$$\mathrm{MSD}\left(\Delta t\right)=\left(4D_{T}+2v^{2}D_{R}^{-1}\right)\tau +\frac{2v^{2}}{D_{R}^{2}}\left(e^{-\Delta t/D_{R}^{-1}}-1\right)$$ 3

In this equation, *D*_T_ and *D*_R_ represent the translational and rotational diffusion coefficients, respectively, *v* is the active particle velocity, and Δ*t* is the elapsed time difference time. The value of *D*_T_ ≈ 0.1 μm^2^ s^–1^ for each cell was determined from the Brownian motion observed in the absence of an electric field, as described above. The rotational diffusion coefficient, *D*_R_, was set to the theoretical value for spherical particles in water at room temperature ( $D_{R}^{\mathrm{th}}=\frac{k_{B}T}{8\pi \eta R^{3}}=0.160$ rad^2^ s^–1^), consistent with the values obtained from mean-squared angular displacement curves for various illumination intensities and electric field strengths (Figure S7).

### Numerical Simulations

The numerical model used for the simulations was previously developed in ref 60 and is based on solving the following equations of motion:

$$\begin{array}{cl} m\ddot{x} & =f_{x}(r,\tau ,\theta )-\gamma_{T}\dot{x}+\sqrt{2k_{B}T\gamma_{T}}\eta_{x}(t)\\ m\ddot{y} & =f_{y}(r,\tau ,\theta )-\gamma_{T}\dot{y}+\sqrt{2k_{B}T\gamma_{T}}\eta_{y}(t)\\ I\ddot{\theta } & =\gamma_{R}\dot{\theta }+\sqrt{2k_{B}T\gamma_{R}}\eta_{\theta }(t) \end{array}$$ 4

where the force acting on the particles is given by

$$\begin{array}{rlll} f_{x}\left(r,\tau ,\theta \right) & =V\left(r,\tau \right)\cos\left(\theta \right)\gamma_{T} & & \\ f_{y}\left(r,\tau ,\theta \right) & =V\left(r,\tau \right)\sin\left(\theta \right)\gamma_{T} \end{array}$$ 5

Here, *m* and *I* are the mass and moment of inertia of the particles (density ρ = 2.5 g cm^–3^, radius *r* = 1 μm). γ_T_ and γ_R_ are the translational and rotational friction coefficients and *V* (**r**, τ) is the response and position-dependent velocity. Finally, η_*x*_(*t*), η_*y*_(*t*), and η_θ_(*t*) are Gaussian white noise terms that fulfill:

$$\begin{array}{rlll} & \left\langle \eta_{x}\right\rangle =\left\langle \eta_{y}\right\rangle =\left\langle \eta_{\theta }\right\rangle =0 & & \\ & \left\langle \eta_{x}^{2}\right\rangle =\left\langle \eta_{y}^{2}\right\rangle =\left\langle \eta_{\theta }^{2}\right\rangle =1 \end{array}$$ 6

To set the maximum particle speeds in the simulations, we used a skewed-normal distribution based on the velocities measured in the most active (illuminated) regions during the experiments. We then calculated the corresponding minimum speeds by applying the experimentally measured speed ratio between illuminated and nonilluminated areas. This ratio was obtained from fits to the MSD curves using eq 3.

## Supplementary Material









## Notes

Experimental procedures for particle fabrication and setup; characterization data including X-ray diffraction (XRD) and rotational diffusion analysis; supplementary data on velocity response to electric field and methanol concentration; and additional simulation results on localization (PDF)
Time-lapse video of a particle ensemble subjected to multiple cycles of global illumination modulation between 6.3W cm^–2^ and 0W cm^–2^, with an applied electric field of Vpp = 5 V at 4 kHz (Video S1) (MP4)
Time-lapse video of a particle ensemble navigating a checkerboard illumination pattern, with alternating illuminated (violet overlay, 6.3W cm^–2^) and nonilluminated (gray) regions (Video S2) (MP4)
Time-lapse video of simulated particle trajectories with spatially varying velocities, based on input data extracted from the experiment shown in Video S2 (see the Methods section for details) (Video S3) (MP4)
