# Supplementary material for: Density Modulations in Active Colloidal Systems through Orthogonal Propulsion Control and Sensory Delays
Source: ACS Nano. 2025 Oct 27;19(45):39210–9. doi: 10.1021/acsnano.5c12596 (PMC12632167; doi:10.1021/acsnano.5c12596)
Supplement: Supplementary file 1 [file nn5c12596_si_001.pdf]

Supporting Information: Density modulations in active colloidal systems through orthogonal propulsion control and sensory delays

Ueli Töpfer, Maximilian R. Bailey, Sanjay Schreiber, Federico Paratore,* and Lucio Isa*

Department of Materials, ETH Zurich, 8093 Zurich, Switzerland

E-mail: federico.paratore@mat.ethz.ch; lucio.isa@mat.ethz.ch

Particle velocity as a function of the electric field

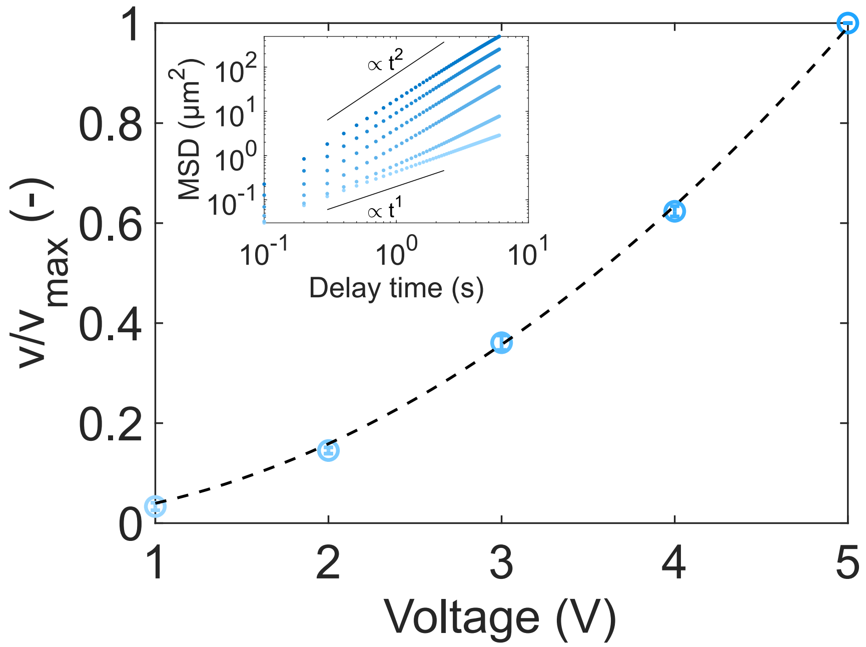

Figure S1: **Normalized particle velocity as a function of the applied potential** Particle velocity increases quadratically with increasing applied voltage. The values were averaged over three measurements obtained at constant illumination intensity of $6.3\,\mathrm{W\,cm^{-2}}$ and AC frequency of $4\,\mathrm{kHz}$ and normalized by the maximum value. The active velocities were calculated from ensemble averaged MSD curves as shown in the inset obtained at a constant illumination intensity of $6.3\,\mathrm{W\,cm^{-2}}$.

Velocity adaptation under switched electric field

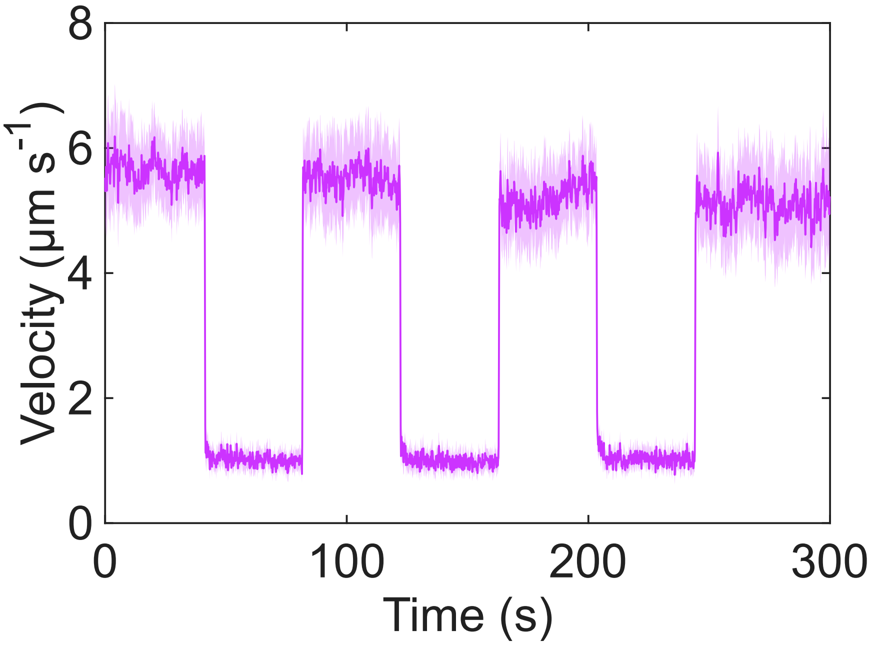

Figure S2: **Velocity adaption under switched electric field** Ensemble-averaged swimming velocity of particles subjected to multiple cyclic changes of the electric field (5 V/0 V, 4 kHz) under constant illumination. The velocities were calculated from the displacement over 300 ms and averaged across the particle ensemble, with the shaded area representing the standard deviation. The illumination intensity was kept constant at 6.3 W cm^{-2}.

Methanol

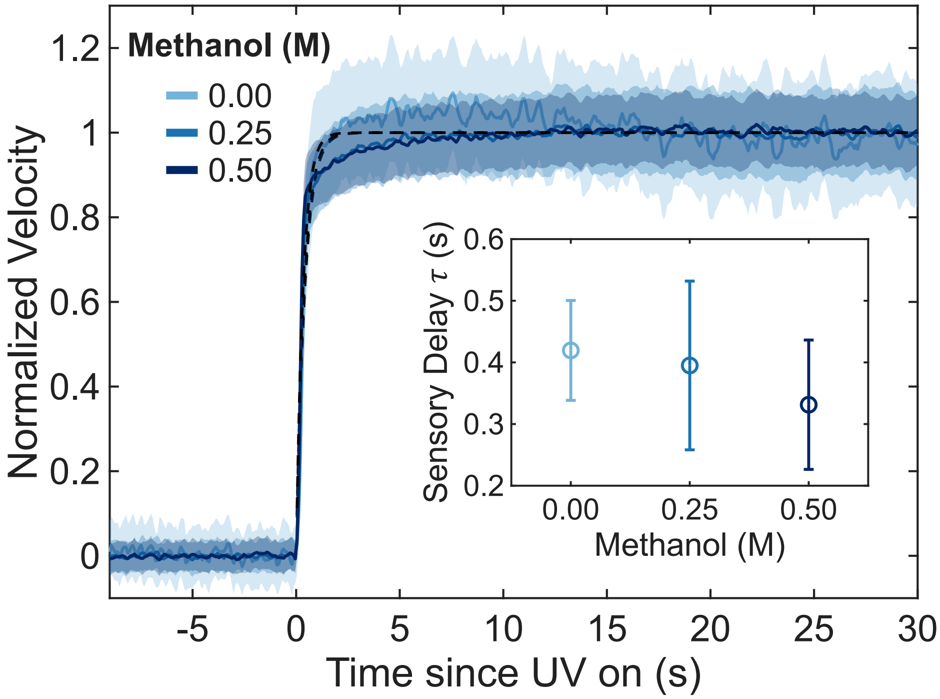

Figure S3: **Influence of methanol on sensory delay.** Mean swimming velocity of particles in pure water (no methanol), and in solutions containing 0.25 M and 0.5 M methanol, showing the increase in velocity following the switch from OFF to ON illumination. For each condition, velocities were first ensemble-averaged per measurement, and then averaged across three measurements per cell from at least three separate cells and particle batches. All experiments were performed at a frequency of 4 kHz, with the voltage amplitude V_{pp} adjusted to ensure a pronounced velocity difference between the illuminated (ON) and non-illuminated (OFF) states. Velocities were calculated from particle displacements over 300 ms. The shaded area represents the standard deviation, obtained via error propagation across the individual measurements. Black lines correspond to exponential fits of the velocity decay, obtained using equation 1 in the main text. The inset shows the mean and standard deviation of the fitted decay times for each condition.

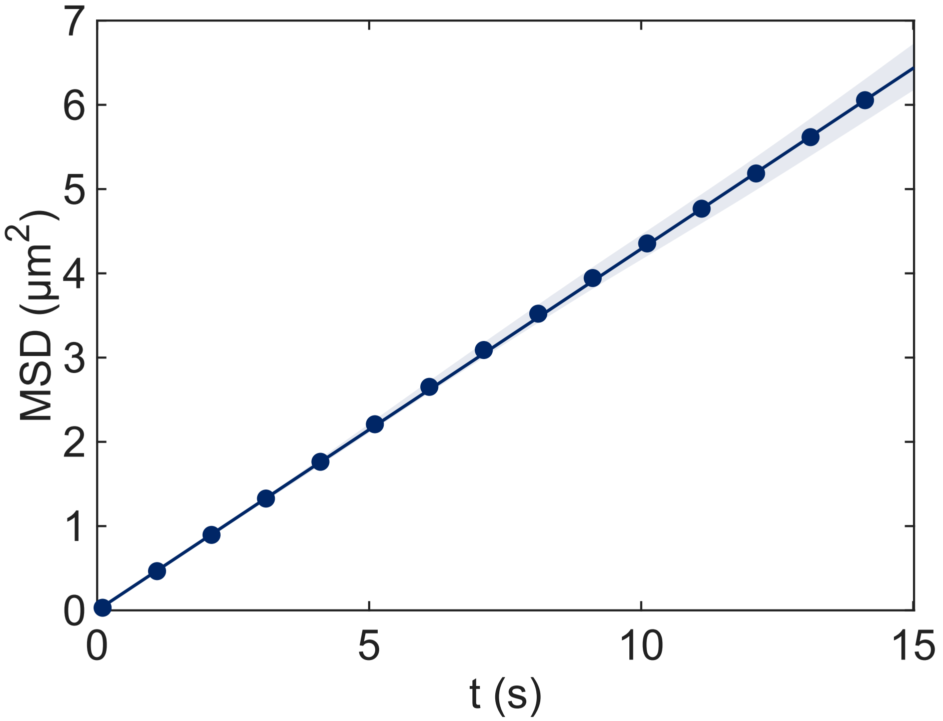

Figure S4: **MSD of particles in presence of methanol without applied AC-field.** Ensemble-averaged MSD of particles under uniform illumination ($6.3\,\mathrm{W\,cm^{-2}}$) in $0.5\,\mathrm{M}$ methanol without an applied electric field. The solid line represents a linear fit, $\mathrm{MSD}(t) = 4D_\mathrm{T}t$, yielding $D_\mathrm{T} = 0.11\,\mathrm{\mu m^2\,s^{-1}}$. The shaded area corresponds to the standard error of the mean.

Average trajectory length

The average trajectory length, L^{eff}, of a particle traversing a square of size L can be determined by integrating over all possible paths where the particle enters through one side and exits through any of the other three sides. This calculation can be decomposed into contributions from trajectories that exit through the opposite side and those that exit through one of the adjacent sides. The latter contribution is accounted for twice, corresponding to exits through either of the two adjacent sides:

$$\begin{aligned} L^{\text{eff}} &= \frac{1}{3}\left(t^{\,\text{top}} + 2\,t^{\,\text{side}}\right) \\ &= \frac{1}{3}\int_0^L \int_0^L \left(\sqrt{(x-y)^2 + L^2} + 2\sqrt{x^2+y^2}\right)\, dx\, dy \approx 0.82\, L \end{aligned} \tag{1}$$

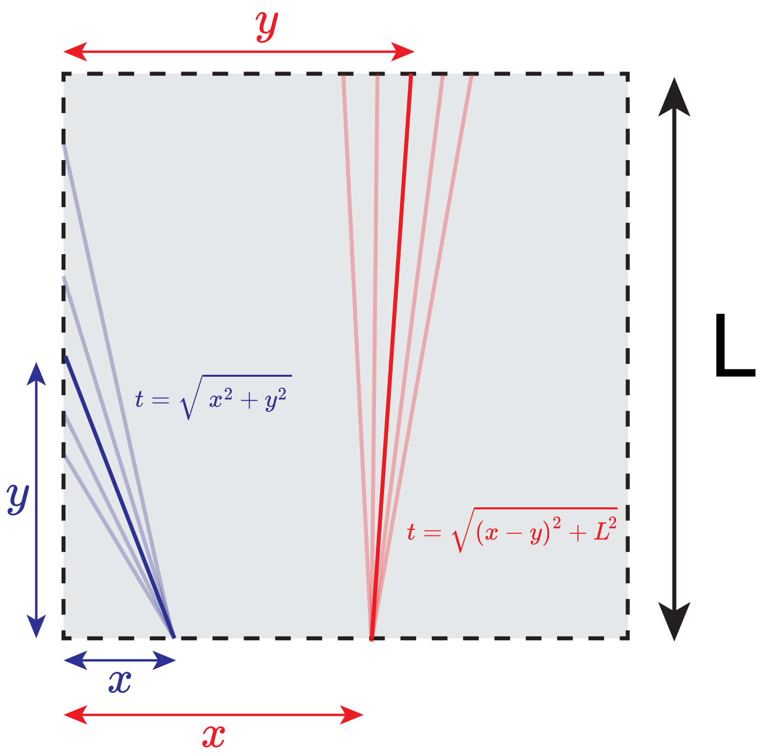

Figure S5: **Average trajectory length through a square** Sketch to visualize the calculation of the length of a trajecotry entering the square and leaving it through the side (blue) or the top (red).

XRD measurement of TiO_2

For the measurement, Ti_3O_5 was deposited on a silicon wafer following the same protocol as for the particle coating (see Methods).

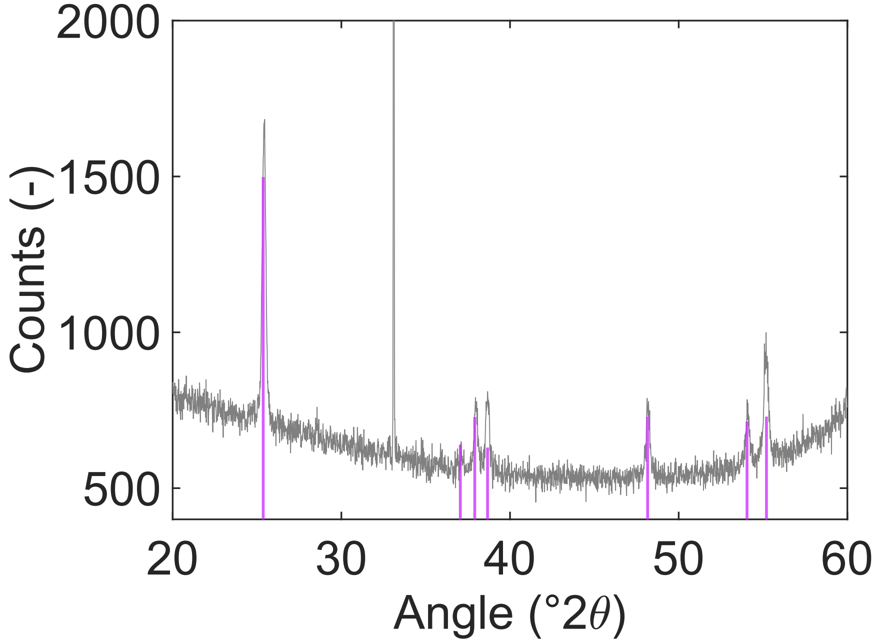

Figure S6: **XRD spectrum of titania** X-ray powder diffraction spectrum of the annealed Ti_3O_5 and reference peaks of anatase titania (American Mineralogist Crystal Structure Database 0010735) shown in gray and violet, respectively. The unlabeled peak at around 33° is an artifact from the Si wafer used as a substrate.[1]

Rotational diffusion under varied voltage and illumination

Values for the rotational diffusion coefficient were extracted from mean squared angular displacement (MSAD) curves by fitting them with $\mathrm{MSAD}(\tau) = 2D_\mathrm{R}\tau$, assuming Brownian dynamics with one rotational degree of freedom. The orientation of the particles was obtained by placing an axis connecting the center of the particle to the center of the cap. The center of the cap was determined in a second image analysis step with a more stringent thresholding.

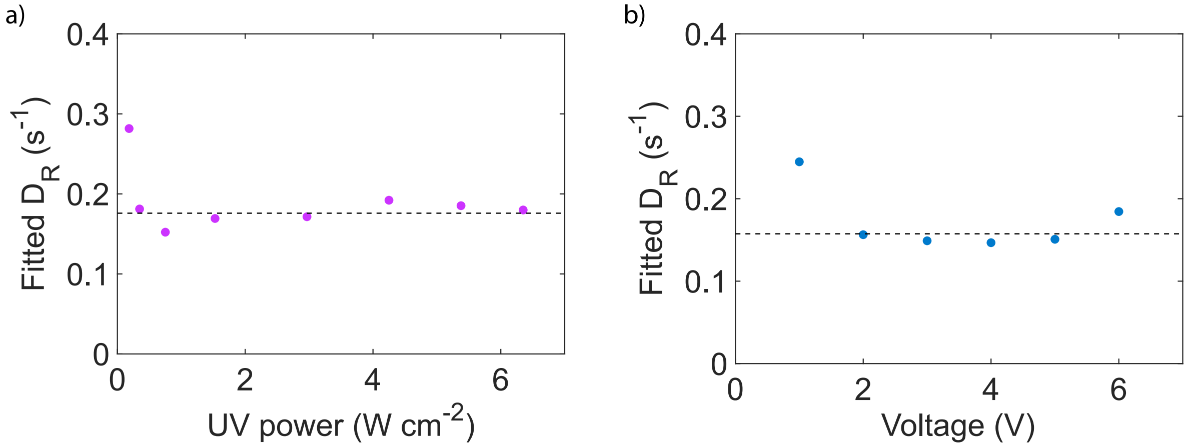

Figure S7: **Influence of illumination and voltage on rotational dynamics** a) Rotational diffusion coefficient D_R as a function of illumination intensity. The dashed gray line indicates the average value of $0.1759\,\mathrm{s}^{-1}$. The voltage was kept constant at 4 V and 4 kHz throughout the experiment. The value at $0.18\,\mathrm{W\,cm}^{-2}$ was excluded from the averaging as a minimum intensity was required to ensure a particle orientation perpendicular to the substrate and thus only one rotational degree of freedom. b) Rotational diffusion coefficient D_R as a function of applied potential. The dashed line corresponds to a mean value of $D_\mathrm{R} = 0.1574\,\mathrm{s}^{-1}$. The illumination intensity was kept constant at $6.3\,\mathrm{W\,cm}^{-2}$ throughout the experiment. The value for a potential of 1 V was excluded with the same reasoning as in a).

Evolution of particle localization

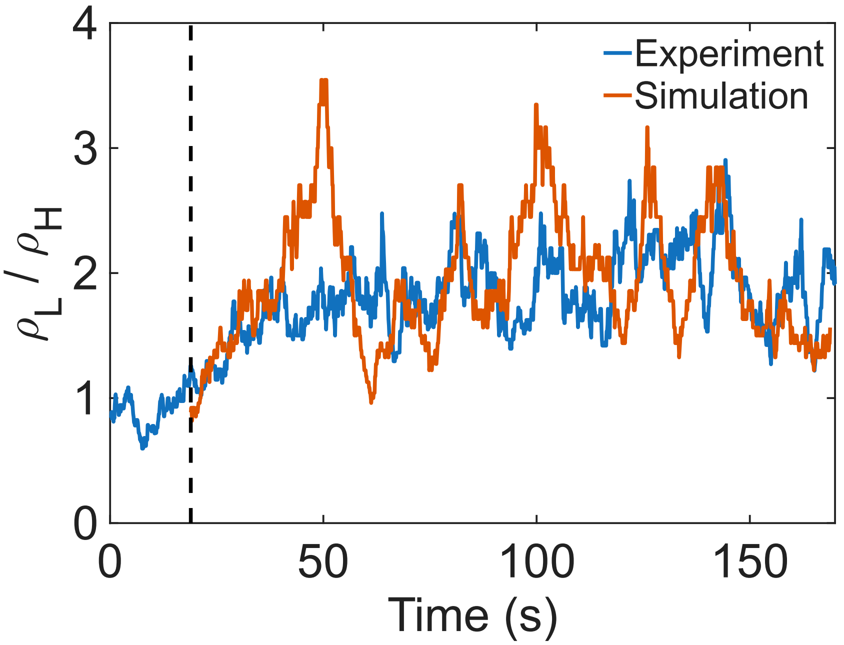

Figure S8: **Time evolution of particle localization.** Temporal evolution of particle localization, quantified as the density ratio between low-velocity (dark, ρ_L) and high-velocity (illuminated, ρ_H) regions. Experimental data were acquired under a projected illumination checkerboard of size $L = 60\,\mu m$ with alternating intensities of $0\,W\,cm^{-2}$ (gray) and $6.3\,W\,cm^{-2}$ (violet), and an applied electric field of 4 V at 4 kHz. The simulation data were obtained from numerical simulations using spatially varying particle velocities extracted from experimental measurements. The dashed line marks the onset of the illumination pattern.

Particle distribution under patterned illumination

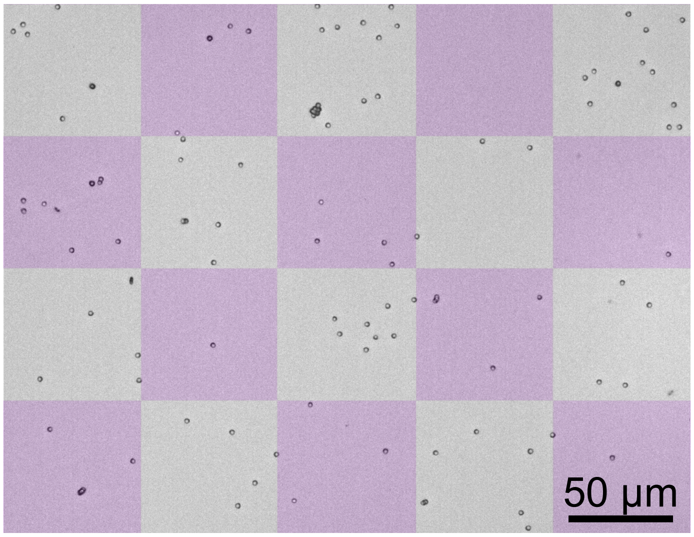

Figure S9: **Spatial particle distribution.** Microscopy image showing the particle distribution within a projected illumination checkerboard of size $L = 60\,\mu\text{m}$, featuring alternating regions of zero ($0\,\text{W}\,\text{cm}^{-2}$, gray) and high ($6.3\,\text{W}\,\text{cm}^{-2}$, violet) intensity. The image was acquired 100 s after illumination onset under an electric field of 4 V and 4 kHz.

Resolution of projected light patterns

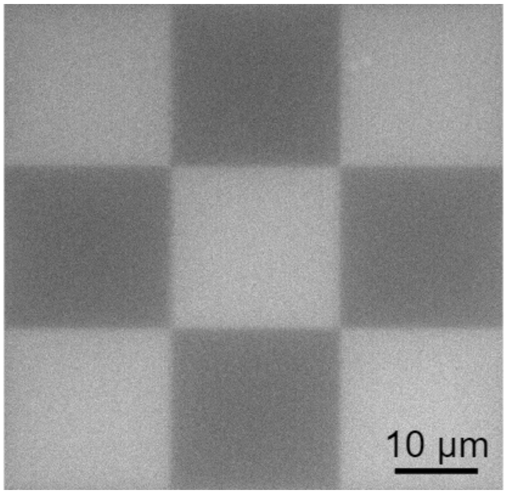

Figure S10: **Projection resolution.** Microscopy image showing the reflection of a 20 μm light pattern at a glass/air interface. The image qualitatively demonstrates the spatial resolution and edge sharpness achievable with the digital micromirror device (DMD).

Quantification of particle localization under increased delay

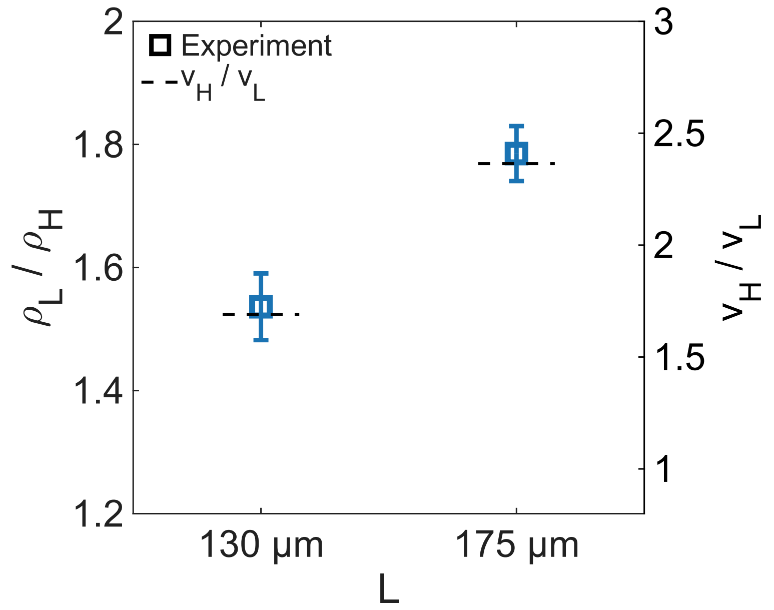

Figure S11: **Quantification of particle localization** Localization, defined as the ratio of particle density in the low- and high-activity areas ρ_L/ρ_H, in experiments with addition of 0.33 M methanol for two different pattern sizes and velocity ratios. Dashed lines represent the experimentally determined velocity ratios v_H/v_L.

Localization under increased velocity contrast

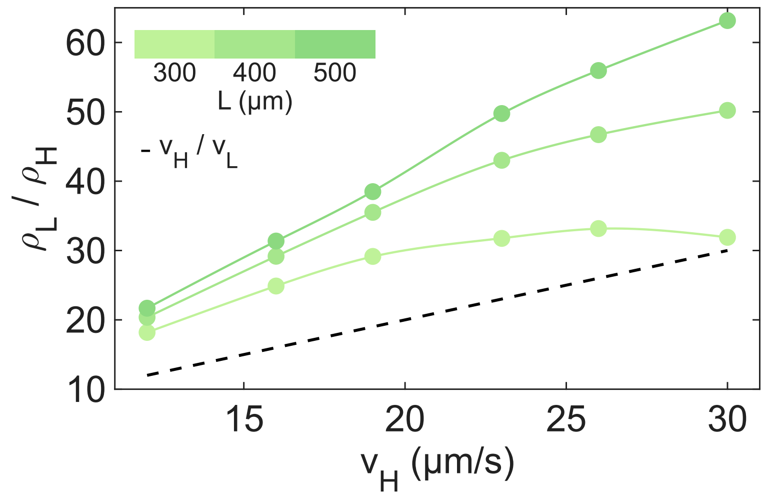

Figure S12: **Localization under increased velocity contrast** Localization ρ_L/ρ_H as a function of v_H for checkerboard patterns with sizes ranging from $L = 300\,\mu\text{m}$ to $500\,\mu\text{m}$. v_L is fixed to $1\,\mu\text{m}\,\text{s}^{-1}$, the sensory delay to $\tau = 8\,\text{s}$ – comparable to the delay with 0.5 M of methanol, the translational diffusivity to $D_\text{T} = 7.76 \times 10^{-2}\,\mu\text{m}^2\,\text{s}^{-1}$, and the rotational diffusivity to $D_\text{R} = 0.0517\,\text{s}^{-1}$ (corresponding to values expected for particles with a radius of $2\,\mu\text{m}$). Continuous lines represent smoothing splines fitted to individual simulations (colored circles).

List of Supplementary Videos

- **Video S1:** Time-lapse video of a particle ensemble subjected to multiple cycles of global illumination modulation between $6.3\,\mathrm{W\,cm^{-2}}$ and $0\,\mathrm{W\,cm^{-2}}$, with an applied electric field of $V_{pp} = 5\,\mathrm{V}$ at 4 kHz. The color bar indicates the particle velocity, calculated from the displacement over a 300 ms time window. The video corresponds to Figure 2 in the main text and is shown at 5× real-time speed.

- **Video S2:** Time-lapse video of a particle ensemble navigating a checkerboard illumination pattern, with alternating illuminated (violet overlay, $6.3\,\mathrm{W\,cm^{-2}}$) and non-illuminated (gray) regions. An electric field of $V_{\mathrm{pp}} = 6\,\mathrm{V}$ at 4 kHz is applied throughout the experiment. The color bar indicates the particle velocity, calculated from the displacement over a 300 ms time window. The video is shown at 8× real-time speed.

- **Video S3:** Time-lapse video of simulated particle trajectories with spatially varying velocities, based on input data extracted from the experiment shown in Video S2 (see Methods section for details). Regions of high particle velocity are shown with a violet overlay, while low-velocity regions appear in gray. The color bar indicates the instantaneous particle velocity, calculated from displacement over a 300 ms time window. The video is shown at 8× real-time speed.

References

(1) Zaumseil, P. High-resolution characterization of the forbidden Si 200 and Si 222 reflections. *Journal of Applied Crystallography* **2015**, *48*, 528–532.
